# Cardio-oncology: management of cardiovascular toxicity

**DOI:** 10.12688/f1000research.14542.1

**Published:** 2019-01-30

**Authors:** Timothy M. Markman, Maurie Markman

**Affiliations:** 1 Department of Medicine, Cardiovascular Division, Hospital of the University of Pennsylvania, University of Pennsylvania, Philadelphia, PA, USA; 2 Cancer Treatment Centers of America at Eastern Regional Medical Center, Philadelphia, PA, USA

**Keywords:** cardio-oncology, cardiovascular toxicity, arrhythmia, heart failure

## Abstract

Traditional chemotherapeutic agents and newer targeted therapies for cancer have the potential to cause cardiovascular toxicities. These toxicities can result in arrhythmias, heart failure, vascular toxicity, and even death. It is important for oncologists and cardiologists to understand the basic diagnostic and management strategies to employ when these toxicities occur. While anti-neoplastic therapy occasionally must be discontinued in this setting, it can often be maintained with caution and careful monitoring. In the second of this two-part review series, we focus on the management of cardiovascular toxicity from anthracyclines, HER2/ErbB2 inhibitors, immune checkpoint inhibitors, and vascular endothelial growth factor inhibitors.

## Introduction

Cardiovascular disease and cancer remain leading causes of mortality in the United States. The co-existence of cardiovascular disease and toxicity that develops as a result of cancer therapy presents a growing challenge for both oncologists and cardiologists. As cancer therapy outcomes continue to improve, this aging population is living long enough to experience adverse effects from therapeutic complications, including electrophysiological abnormalities, vascular toxicity, and heart failure (HF).

These toxicities and their proposed and established mechanisms were discussed in detail in the first installment of this review ^1^. Cardiovascular complications often represent a limiting factor in the therapeutic options for many otherwise treatable and potentially curable malignancies. It is essential that oncologists and cardiologists be aware of the most up-to-date strategies for management in order to optimize the potential for further therapy. In this review, we explore the management of select cardiovascular toxicities from anthracyclines, HER2/ErbB2 inhibitors, immune checkpoint inhibitors, and vascular endothelial growth factor (VEGF) inhibitors.

## Anthracyclines

Cardiomyopathy from anthracyclines, initially daunorubicin, was the first described cardiotoxicity ^2^. The risk is dose dependent, with over 25% of patients experiencing clinical HF with cumulative doses of 550 mg/m ^2^ or greater. Subclinical evidence of toxicity is present in 30% of patients, even at cumulative doses of 180 to 240 mg/m ^2^ ^3, 4^. While clinicians must be aware that higher doses are associated with increased risk, it is also important to recognize that there is no dose at which the risk for cardiac toxicity is zero ^3^.

It is imperative to recognize any pre-existing cardiac dysfunction in patients prior to the initiation of therapy ^5^. At a minimum, this includes careful clinical evaluation and assessment of traditional cardiovascular risk factors including the presence of coronary artery disease, diabetes mellitus, hypertension, or smoking. Given the effect on left ventricular systolic function, this should be assessed prior to beginning therapy ^6^. Most commonly, a transthoracic echocardiogram is the screening test of choice. Multigated acquisition radionuclide ventriculography or cardiac magnetic resonance imaging can be used, although these techniques are limited by radiation exposure and cost/availability, respectively. Any evidence of pre-therapy reduced ejection fraction should prompt referral to a cardiologist for evaluation and optimization prior to anthracycline exposure.

Once therapy is initiated, monitoring strategies must be individualized to identify early evidence of cardiotoxicity. Echocardiography is often recommended at 3-month intervals with additional imaging mandated by clinical evidence of HF ^7^. Cardiotoxicity in this setting has been defined by a decline in ejection fraction by ≥10% (e.g. from 50% to 40%) or ≥5% in the presence of HF symptoms, although a variety of criteria have been used ^4^. Advanced imaging strategies have been developed to identify early myocardial dysfunction with increased sensitivity and specificity. This includes multiple techniques to characterize myocardial strain, which appears to be very sensitive. A reduction of 10–15% in global longitudinal strain is the most useful parameter to identify the early development of anthracycline-induced cardiomyopathy ^4, 8^. Cardiac biomarkers (especially troponin and brain natriuretic peptide) are associated with the development of left ventricular dysfunction and symptomatic HF; although their routine use as screening tests is promising, there is not universal agreement on the significance of isolated biomarker elevation ^4, 8^. Prolongation of the QT interval on electrocardiogram has also been shown to be associated with the development of anthracycline-induced ventricular dysfunction ^9^.

Based on the mechanisms discussed in the first part of this review, several therapeutic strategies have been proposed to treat anthracycline-induced cardiotoxicity or to prevent it in high-risk patients. Prevention involves avoidance of anthracyclines when possible, utilization of minimal cumulative doses, and preference for continuous infusion versus bolus dosing ^10^. A PEGylated liposomal version of doxorubicin has been shown to decrease the circulating concentration of free doxorubicin without decreased effectiveness, although high cost has limited its utility ^11^.

Dexrazoxane is protective against anthracycline cardiotoxicity by mediating topoisomerase 2β. Its effectiveness has been well established in numerous clinical trials, although two controversies limit its widespread adoption ^4, 12^. There was initial evidence that the use of dexrazoxane decreased anti-neoplastic efficacy and although this did not hold up in meta-analysis, the U.S. Food and Drug Administration has approved its use only in patients who have already received at least 300 mg/m ^2^ of doxorubicin for metastatic breast cancer ^12, 13^. In a pediatric population receiving simultaneous etoposide and doxorubicin, there is evidence of an increased risk for secondary malignancies, potentially due to genetic instability ^14^. While this association has not been clearly established, the signal must be taken seriously in this population. Use of dexrazoxane should be considered given its established efficacy and safety exclusive of patients receiving simultaneous etoposide.

Once clinical HF or imaging evidence of reduced left ventricular systolic function is present, treatment should follow established guidelines for patients with non-ischemic cardiomyopathy. The mainstays of therapy are angiotensin-converting enzyme (ACE) inhibitors and beta-blockers uptitrated to the maximum tolerated doses with diuretics as needed ^15, 16^. The results of the PRADA trial have suggested that treatment with candesartan during anthracycline therapy for early breast cancer provides protection against early decline in left ventricular function ^17^. Newer agents, including sacubitril/valsartan, and advanced therapies for HF should be considered when necessary. Because anthracycline-induced cardiomyopathy is considered irreversible, the development of reduced LV function has been recommended as a contraindication to continuing anthracyclines, although cessation of lifesaving therapy should be considered only as a last resort ^4^.

The role of these agents and HMG-CoAr inhibitors (statins) for the prevention of anthracycline-induced cardiomyopathy remains unclear though is currently under investigation in the Preventing Anthracycline Cardiovascular Toxicity With Statins (PREVENT) trials, among others. The impact of physical activity has not been conclusively established from clinical studies, although animal models and evidence from other non-ischemic cardiomyopathies suggest it may be beneficial ^18^.

## HER2/ErbB2 inhibitors

Trastuzumab, a monoclonal antibody directed against HER2/ErbB2 receptors, has also been established as a cause of left ventricular dysfunction. This agent is used for the treatment of breast cancers that overexpress HER2/ErbB2 and is known to cause a reversible cardiomyopathy that is otherwise phenotypically similar to anthracycline-induced cardiomyopathy. The symptomatic HF that develops can be severe and life threatening, though it is generally mild and improves with medical management and discontinuation of therapy. Trastuzumab can often be resumed after recovery of ventricular function ^4^.

Recommendations for pre-therapy screening and imaging studies are generally similar to those discussed above for anthracyclines. Patients at high risk should ideally avoid concurrent treatment with anthracyclines, which results in worse cardiovascular outcomes ^19^. Given the reversibility of trastuzumab-induced left ventricular dysfunction, a more lenient cut-off of ejection fraction <40% to withhold therapy has been proposed. There is considerable evidence that strain is strongly predictive of trastuzumab-associated cardiotoxicity and is even more specific when combined with elevated troponin ^20^. A monitoring regimen of troponin measurement at baseline and every 3 weeks with echocardiography with strain every 3 months has been suggested ^6^. When left ventricular dysfunction or HF does develop, institution of targeted therapy based on the guidelines discussed above is recommended without substantial evidence in this population.

## Immune checkpoint inhibitors

By allowing the immune system to upregulate its activity against malignant cells, immune checkpoint inhibitors have proven to be highly effective cancer drugs. Cardiotoxicity, which can include arrhythmias, heart block, and myocarditis, is due to autoimmune activity against normal myocardium ^21^. Although the true incidence is unknown, it has been increasingly recognized and reported, especially when combination therapy is used ^22^.

The gold standard of diagnosis remains myocardial biopsy, although the development of heart block, ventricular arrhythmias, left ventricular dysfunction, or clinical HF after the initiation of immune checkpoint inhibitors is generally considered sufficient to initiate treatment. Additional diagnostic studies, especially cardiac magnetic resonance imaging to identify myocarditis, have been suggested. There are no established guidelines and there is very little evidence to guide the management of cardiotoxicity when it occurs. Based on the autoimmune mechanism of toxicity, immunosuppressive agents are recommended. High-dose corticosteroids have been used with some success and when insufficient alternative immunosuppressive regimens have been utilized, including mycophenolate mofetil and tumor necrosis factor-alpha antagonists ^23– 25^. These regimens come from more established management of other autoimmune toxicities with immune checkpoint inhibitors including hepatitis, pneumonitis, colitis, and endocrinopathies. Therapy may be withheld in the acute setting, but resumption should be considered if ventricular dysfunction resolves.

There are no clear guidelines for prevention or monitoring, and a deeper understanding of the pathophysiology is needed before recommendations can be established. Physicians must be alert to this important complication, and further research and establishment of guidelines is mandatory.

## Vascular endothelial growth factor inhibitors

Several malignancies, including renal cell carcinoma and hepatocellular carcinoma, can be treated with VEGF inhibitors. Their use can be limited by both left ventricular dysfunction and, more commonly, hypertension. Although the majority of cardiovascular complications are not fatal, they are associated with significant morbidity and can impact the ability to continue potentially lifesaving therapy ^26^. For this reason, early recognition and management are essential.

Approximately a quarter of patients experience asymptomatic decline in left ventricular function, while symptomatic HF is seen in 4–8% ^27, 28^. Hypertension, however, has been reported in nearly half of patients with varying clinical significance depending on the baseline blood pressure and comorbid conditions ^27, 29, 30^. In the most recent meta-analysis of 77 studies, severe hypertension is noted in 7.4% of patients, arterial thromboembolism in 1.8%, myocardial ischemia in 1.7%, and ventricular dysfunction in 2.3% ^31^.

Hypertension secondary to kinase modification can be marked and result in significant downstream effects on the cardiovascular system. Even without end organ damage, poorly controlled blood pressure often results in the discontinuation of essential anti-neoplastic therapy ^32, 33^. Diagnosed based on simple ambulatory blood pressure screenings, hypertension occurring within the first month after treatment initiation is likely to be secondary to therapy. While there are many antihypertensive medications that can be used, ACE inhibitors are considered first line owing to favorable hemodynamic and anti-proteinuric effects and evidence of improved mortality ^34^. When other agents are necessary, attention should be paid to potential drug–drug interactions given the effects of VEGF inhibitors, especially sorafenib, or the cytochrome P450 system. Although hypertension in this setting can have significant consequences, providers should resist unnecessary discontinuation because an increase in blood pressure may be a sign of efficacy of VEGF inhibitor therapy ^35^. **

Patients with pre-existing left ventricular dysfunction or coronary artery disease must be monitored closely and treated with the same strategies discussed for anthracyclines and HER2/ErbB2 inhibitors ^27^ *.* The mechanism of HF, however, is generally felt to be secondary to hypertension and increased afterload in vulnerable patients. For that reason, the main focus is on the prevention and treatment of severe hypertension ^36^. Other cardiovascular toxicities that have been noted in association with VEGF inhibitors include venous and arterial thrombosis, coronary artery disease, acute coronary syndromes, and conduction abnormalities ^4^. These complications should be managed according to their respective current guidelines with additional consideration for cessation or modification of therapy.

## Conclusion

The field of cardio-oncology has rapidly expanded, but there remains significant room for growth in research and clinical practice. This two-part series highlights only a subset of the agents capable of causing profound cardiovascular toxicities, and many complications of cancer and cancer therapy were not discussed, including pericardial disease, myocardial ischemia, and QT prolongation. Numerous questions are unanswered about the mechanism of toxicities and the optimal screening and management strategies. Given the tremendous complexity of impacted patients, close communication and collaboration between oncologists and cardiologists is mandatory at all stages of therapy. Oncologists must know when to turn to cardiologists for assistance in risk stratification and management of toxicities when they develop. Critically, cardiologists need to recognize that their role is to facilitate the delivery of lifesaving cancer therapy through optimization and management of toxicity.
